# Items

**Published:** 1879-09

**Authors:** 


### Items.

AMERICAN PUBLIC HEALTH ASSOCIATION. — As it may interest some of our readers, we insert the following notice [ED.]:

*To Members of the American Public Health Association:*

GENTLEMEN: — At a meeting of the Executive Committee, held in Washington, January 3, 1879, it was decided that the principal subject for discussion at the next annual meeting of the Association, to be held in Nashville, Tenn., 18th–21st November, shall be the sanitary condition of cities and towns, especially those of the Southern States.

In selecting a subject of so wide a scope, the committee considers that it will be expedient, if not indeed indispensible to the attainment of useful results, to limit the inquiry to certain specified branches of the general subject rather than to attempt to cover the entire ground of city sanitation.

A few years ago a committee appointed by the Association prepared a series of elaborate schedules of questions for facilitating such an inquiry, which have been recently published in pamphlet form as Circular No. 2, of the National Board of Health. A copy of this pamphlet will be furnished to any member of the Association who desires to take a part in the proposed discussion, on application to Dr. T. J. Turner, secretary of the National Board of Health and a member of the Executive Committee of this Association. His address is "Office National Board of Health, Washington, D. C."

The Executive Committee recommends the following subjects of inquiry: Water Supply, schedule C; Drainage and Sewerage, schedule D; Disposal of Garbage and Excreta, schedule H; Slaughter Houses and Abattoirs, schedule K; Public School

Buildings, schedule M; Public Health Laws, Regulations, etc., schedule R; Expenses of Municipal Sanitation, schedule U.

Attention is also called to the following resolution presented by Dr. A. Gihon, U. S. N., at the last annual meeting:

"Resolved, that the Executive Committee be directed to provide for the investigation and discussion at the next annual meeting, of the most effective means for preventing the spread of venereal disease."

Members who propose to consider these subjects, or who may desire to treat exhaustively some special subject of their own selection, are invited to prepare papers not to require more than thirty minutes in reading, and to forward titles and abstracts in accordance with Sec. VIII of the Constitution.

J. L. CABELL,
*President American Public Health Association,*
*and Chairman of Executive Committee.*

UNIVERSITY OF VIRGINIA,
CHARLOTTESVILLE, Aug. 15, 1879.

EDUCATIONAL PROGRESS. — The *Medical Record*, of New York, for August 23d, says: "In accordance with a long cherished plan, the medical department of Yale College announces a graded course of instruction extending over three years." A fair standard of preliminary education is also required before admission. Thus the good work progresses. But when will the *Medical Record* announce that the New York medical schools have followed so good an example?

PROF. WM. H. BYFORD, M.D., has just returned from Europe. He went abroad to read a paper, by special invitation, before the British Medical Association, at Cork. He selected as his subject, "The Action of Ergot in Intra-Uterine Fibroids."

PROF. JAS. NEVINS HYDE, M.D., is absent from the city, attending a meeting of the American Dermatological Society, and in the next number of the JOURNAL will doubtless give our readers the benefit of his visit.

